# Application of Asymmetric IRT Modeling to Discrete-Option Multiple-Choice Test Items

**DOI:** 10.3389/fpsyg.2018.02175

**Published:** 2018-11-12

**Authors:** Daniel M. Bolt, Sora Lee, James Wollack, Carol Eckerly, John Sowles

**Affiliations:** ^1^Department of Educational Psychology, University of Wisconsin-Madison, Madison, WI, United States; ^2^Educational Testing Service, Princeton, NJ, United States; ^3^Ericsson, Inc., Santa Clara, CA, United States

**Keywords:** item response theory (IRT), computerized testing, multiple-choice, latent ability estimates, Samejima's logistic positive exponent (LPE) model

## Abstract

Asymmetric IRT models have been shown useful for capturing heterogeneity in the number of latent subprocesses underlying educational test items (Lee and Bolt, 2018a). One potentially useful practical application of such models is toward the scoring of discrete-option multiple-choice (DOMC) items. Under the DOMC format, response options are independently and randomly administered up to the (last) keyed response, and thus the scheduled number of distractor response options to which an examinee may be exposed (and consequently the overall difficulty of the item) can vary. In this paper we demonstrate the applicability of Samejima's logistic positive exponent (LPE) model to response data from an information technology certification test administered using the DOMC format, and discuss its advantages relative to a two-parameter logistic (2PL) model in addressing such effects. Application of the LPE in the context of DOMC items is shown to (1) provide reduced complexity and a superior comparative fit relative to the 2PL, and (2) yield a latent metric with reduced shrinkage at high proficiency levels. The results support the potential use of the LPE as a basis for scoring DOMC items so as to account for effects related to key location.

## Application of asymmetric IRT modeling to discrete-option multiple-choice test items

Most item response theory (IRT) models used in educational and psychological measurement contexts assume item characteristic curves (ICCs) that are symmetric in form, implying the change below the inflection point is a mirror image of change above the inflection point (Embretson and Reise, 2000; De Ayala, 2009). Popular examples include the Rasch model, as well as, two-parameter and three-parameter logistic and normal ogive models. While such models are commonly used and have statistical appeal, they arguably provide a poor reflection of psychological response process (van der Maas et al., 2011). Models that attend only to item difficulty and discrimination may miss other important aspects of item heterogeneity, in particular item complexity, a feature that tends to emerge in the form of ICC asymmetry (Samejima, 2000; Lee and Bolt, 2018a). Where heterogeneity related to item complexity is present, more general models may be needed to provide the level of fit required in support of the latent metrics used in reporting scores (Bolt et al., 2014). Asymmetric IRT models have often been found to provide a superior fit to educational test items, including items of both multiple-choice and constructed response formats and of varying content areas (Bazán et al., 2006; Bolfarine and Bazán, 2010; Molenaar, 2015; Lee and Bolt, 2018a,b); thus, greater attention to these models appears warranted.

One context in which asymmetric models may find particular value is in the scoring of discrete option multiple-choice (DOMC) items (Foster and Miller, 2009). The DOMC item format was developed as a variation on the traditional multiple-choice format as a way of minimizing the effects of testwiseness on item performance, while also helping to protect item security through administration of fewer overall response options to examinees (Kingston et al., 2012). The format is becoming increasingly popular with the growing use of computer-based assessments (Willing et al., 2014; Papenburg et al., 2017). Under DOMC, the display of an item stem is followed by a sequence of randomly administered response options. The examinee responds to each option independently (as “correct” or “incorrect”) according to whether or not the response matches the item stem. In typical DOMC administrations, the options are administered until either (a) the examinee endorses a distractor option or fails to endorse a keyed response or (b) all of the keyed responses have been correctly endorsed. If (a) occurs, the item is scored incorrect, while if (b) occurs, the item is scored as correct (Commonly, an additional response option will be administered with some non-zero probability after the final keyed response is shown so that the examinee is not made aware of having seen a keyed response). As the administration of response options is random, not only the number of options but also the order and specific options (in the case of distractor options) administered will vary across examinees. While many DOMC items may contain only one keyed response, others may contain multiple keyed responses. A test of U.S. History, for example, might include an item “Is the following freedom protected by the First Amendment of the Constitution?” followed by the sequential presentation of options “Freedom of the Press,” “Freedom from Unreasonable Searches and Seizures,” “Freedom to Keep and Bear Arms,” “Freedom of Speech,” in which case two of the options (“Freedom of the Press” and “Freedom of Speech”) are keyed responses. In all cases, respondents are scheduled to be administered response options up through the last of the keyed responses; the overall item is scored as correct only if all keyed options are endorsed (and any and all distractor options administered along the way are correctly rejected). Further, in current practice, examinees only receive an overall correct or incorrect score on each item, regardless of the number of options administered to arrive at that outcome.

It has been previously demonstrated that administering the same items in a DOMC format as opposed to a traditional MC format often increases item difficulty (Eckerly et al., 2017). More critical to this paper is the effect of the randomized location of the keyed responses, as this randomization implies that the same item will be administered with more scheduled distractor options (and thus require a correct rejection of more distractors) for some examinees than others. Eckerly et al. (2017) observed that as the number of overall scheduled response options for the item increases, both the difficulty and discrimination of the item tends to increase. From an equity perspective, the increase in difficulty makes it disadvantageous to be administered an item with a later key location (implying a larger number of scheduled response options). Although we can expect the random selection of key locations across items to balance these effects to some extent, this assurance comes only for a sufficiently long test. It is common to see rather large differences emerge for certain individual examinees who by chance received a disproportionate number of items with either a low or high number of scheduled response options (Eckerly et al., 2017).

One way of rectifying this problem is to score DOMC item responses in a way that accounts for the psychometric effects of key location. A natural approach would be to use a suitable IRT model as a basis for such scoring. One contribution of this paper is to show how an asymmetric IRT model, namely Samejima's (2000) logistic positive exponent (LPE) model, can be applied to address such effects. Part of the appeal of the LPE in this context is its potential to simultaneously account for changes in both difficulty and discrimination using fewer parameters than would be required if using the 2PL to account for such effects. For reasons we explain later in the paper, we also argue that the LPE has greater psychological plausibility in such contexts given the conjunctive interaction that occurs between the individual option responses in determining an overall correct response to the item (i.e., an item is scored correct only if all presented distractors are rejected and all keyed options are endorsed).

A second contribution of the paper relates less to DOMC, and more to the opportunity the DOMC application provides in demonstrating the meaningfulness in studying ICC asymmetry from an item validation perspective. Lee and Bolt (2018a), for example, illustrated through simulation how estimates of asymmetry parameters can inform about the *complexity* of a test item, there defined in terms of the “number” of conjunctively interacting response subprocesses (i.e., “steps”) underlying a correct item score (Samejima, 2000). Specifically, items involving a larger number of conjunctively interacting subprocesses were found to have more positive asymmetry in their ICCs. The DOMC format can be viewed as a natural experiment in which the number of conjunctively interacting subprocesses (i.e., viewing the response to each presented option as another subprocess) is systematically manipulated. The potential to extend the results of Lee and Bolt (2018a) to real test data would be an important additional step in supporting the use of asymmetric IRT models for validation purposes, and provide a real-world illustration of how the asymmetry parameter connects to known item complexity.

The remainder of this paper is organized as follows. First, we review the LPE model of Samejima (2000) that would appear applicable to items administered under the DOMC format. We next present a real dataset to which the models can be applied, and demonstrate the observation of ICC asymmetry in comparing the LPE model to other IRT models that similarly attend to the effects of the scheduled number of response options. Finally we demonstrate the implications of the asymmetry on the metric properties of the IRT analysis and offer thoughts for future research.

## Asymmetric IRT models

Although there exist alternative IRT models that introduce asymmetry (Bazán et al., 2006; Molenaar, 2015), in the current analysis we focus on Samejima's (2000) LPE model. The LPE extends the two-parameter logistic model by introducing an item exponent/acceleration parameter; the presence of this exponent parameter results in asymmetric ICCs. Under an LPE model, an item is thus parameterized not only by the *a* and *b* (and possibly *c*) parameters of traditional IRT models, but also an exponent parameter ξ (>0). We then view the probability of successful execution of a single component subprocess (e.g., a response to a single option presented for a DOMC item) on an item *i* by examinee *j* as corresponding to a 2PL model, i.e.,

(1) $$\begin{array}{l} {\text{Ψ}}_{i}\left(\theta_{j}\right)=\;\frac{exp\left[a_{i}\left(\theta_{j}-b_{i}\right)\right]}{1+exp\left[a_{i}\left(\theta_{j}-b_{i}\right)\right]}\;, \end{array}$$

where the slope *a*_*i*_ reflects the discrimination of item *i* and the *b*_*i*_ reflects its difficulty. Then the resulting probability of a correct response to the item under an LPE is written as

(2) $$\begin{array}{l} P\left(U_{ij}=1|\theta_{j}\right)=\;{\left[{\text{Ψ}}_{i}\left(\theta_{j}\right)\right]}^{\xi_{i}}\;. \end{array}$$

In more general measurement contexts, a subprocess, Ψ_*i*_(θ_*j*_), might be viewed as a single step taken in solving an item, such that an item is answered correctly only if all steps are successfully executed. An example might be a long division problem, in which the repeated sequential steps of division, multiplication, subtraction and bringing down the next digit are each thought of as a separate subprocess. As an exponent parameter, ξ could be viewed as defining the “number” of conjunctively interacting subprocesses that underlie an item, and is referred to as an item complexity parameter. Thus, the asymmetry of the ICC depends on how many conjunctive or disjunctive subprocesses are involved in solving the item.

**Figure 1:**
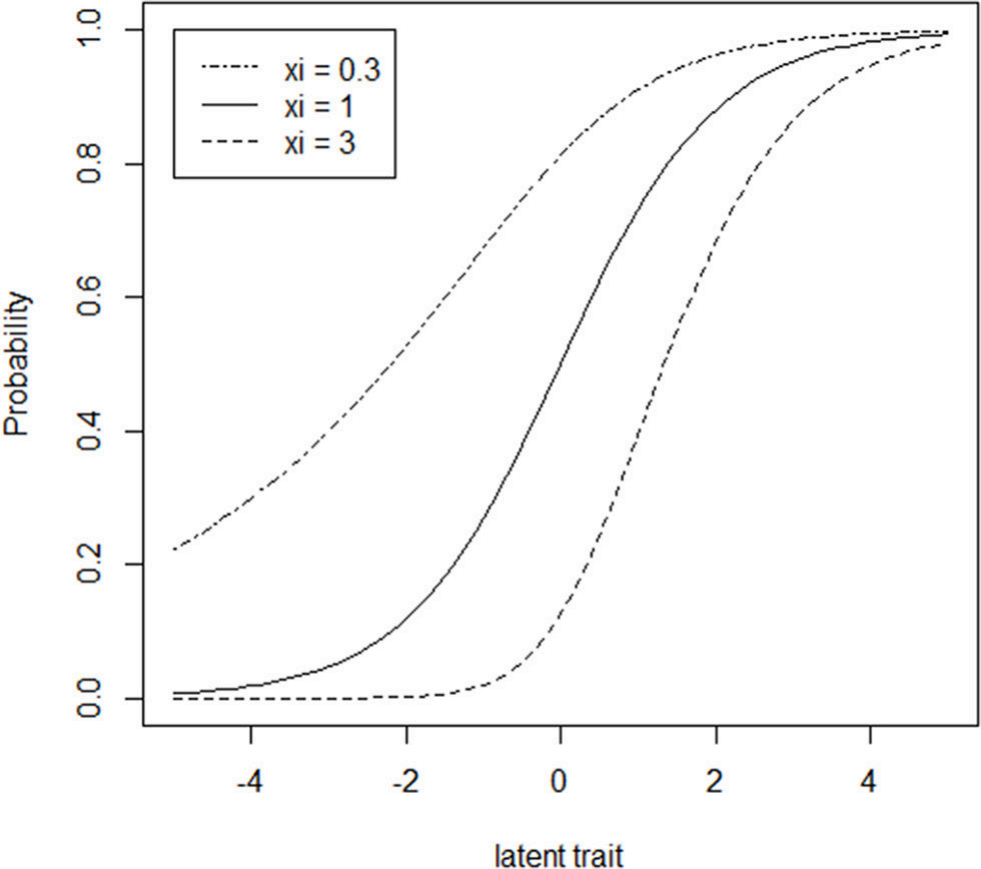
Item characteristic curves (ICCs) for three hypothetical LPE items (a = 1, *b* = 0 for all items) that vary with respect to ξ.

Figure 1 provides an illustration of ICCs for LPE items in which the ξ parameter is varied at levels of 0.3, 1, and 3 while the subprocess parameters, *a* and *b*, are held constant at values of 1 and 0, respectively. When ξ = 1, the ICCs are symmetric as in the traditional two parameter IRT models. When 0 < ξ < 1 (corresponding to multiple disjunctively interacting subprocesses), the ICC accelerates at a faster rate to the right of the inflection point (i.e., the latent trait location at which the slope of the ICC begins decreasing) than it decelerates to the left of the inflection point (Samejima, 2000). In the case of ξ > 1 (corresponding to multiple conjunctively interacting subprocesses), the asymmetric ICC decelerates at a slower rate to the right of the inflection point than it accelerates to the left of the inflection point. As is apparent from Figure 1, ξ also has an influence on the psychometric difficulty and discrimination of the item. Specifically, increases in ξ correspond to increases in both difficulty and discrimination.

As noted earlier, the emphasis in the LPE on conjunctively interacting item subprocesses seemingly provides a close fit to DOMC items, and in particular, the effect related to varying the number of scheduled response options. In particular, the LPE exponent parameter provides a convenient way of accounting for the simultaneous increase in both difficulty and discrimination associated with an increase in the number of response options. Such a phenomenon can be easily captured by the LPE model in (1) and (2) by allowing the ξ parameter to vary in relation to the number of scheduled response options. In particular, for a fixed item we expect ξ to become larger as the number of scheduled response options increases.

## Information technology (IT) certification data

The item response data in the current study come from an information technology (IT) certification test used for employment decisions. The test is administered internationally and primarily to respondents with a college education; the current sample consisted primarily of respondents in Asian countries. We consider data from two forms of the test administered in 2016–2017, each containing 59 items, but with an overlap of 35 items across forms, implying a total of 83 distinct items in the dataset. This data structure permits a concurrent calibration of both forms against a common latent metric. Most (54) of the items had a single keyed response option; however, 24 had two keyed responses, and 5 had three keyed responses. The items with three keyed responses had a maximum of five response options, while all other items had a maximum of four response options. A total of 648 examinees provided item response data. All DOMC items were adapted from items originally administered using a traditional multiple-choice format. As examinees must respond to each computer-administered response option to proceed to the next option/item, there were no missing data.

As is typically the case with DOMC items, each administered item is scored correct/incorrect on this IT test regardless of the number of response options administered. Although this form of item scoring is straightforward, it might be viewed as suboptimal to the extent that an item will naturally be more difficult when scheduled with more response options than with fewer. Table 1 shows for each item category how the *p*-values and item-total correlations change by the scheduled number of response options across all DOMC items in the IT data. Based on these observations, it would seem appropriate to reward correct responses following a larger number of scheduled response options. Such a form of scoring can be accommodated using IRT models provided a suitable accounting for the effect of number of scheduled response options. We consider several such models in the next section.

**Table 1:** Mean classical item difficulties (*p*-values) and discriminations (item-total correlations) as a function of the number of scheduled response options, single keyed, double keyed, and triple keyed items.

	**Single keyed items (*****N*** = **54)**		**Double keyed items (*****N*** = **24)**		**Triple keyed items (*****N*** = **5)**	
**# Scheduled Resp options**	***p*-value**	**Item-total corr**	***p*-value**	**Item-total corr**	***p*-value**	**Item-total corr**
1	0.64	0.22				
2	0.49	0.31	0.47	0.21		
3	0.37	0.38	0.32	0.31	0.34	0.35
4	0.31	0.40	0.22	0.34	0.26	0.38
5					0.19	0.35

## Models for DOMC item response

Various IRT models could be considered to account for the psychometric effects of the scheduled number of response options on item functioning. One possibility is to fit a 2PL model in which the *a* and *b* parameters of the model vary as a function of the scheduled number of response options. Assuming *k* denotes the scheduled number of response options, the model can be written as:

(3) $$\begin{array}{l} P\left(U_{i\left(k\right),j}=1|\theta_{j}\right)=\;\frac{exp\left[a_{i\left(k\right)}\left(\theta_{j}-b_{i\left(k\right)}\right)\right]}{1+exp\left[a_{i\left(k\right)}\left(\theta_{j}-b_{i\left(k\right)}\right)\right]}\;, \end{array}$$

where *i(k)* refers to item *i* under a condition in which *k* response options are scheduled. In the model shown in (3), we effectively treat each item having a different number of scheduled response options as an independent item, thus having its own separately estimated discrimination and difficulty parameters. As the effects of the number of response options are most pronounced with respect to difficulty, we also consider a special case in which only the difficulty (*b*) parameter varies with respect to response options, effectively constraining *a*_*i*(*k*)_ = *a*_*i*_ for all *k*. As an even more restrictive model, we also consider a special case in which both *a*_*i*(*k*)_ = *a*_*i*_ and *b*_*i*(*k*)_ = *b*_*i*_, effectively assuming no effect of *k* on the 2PL item parameters. In our comparison of models to the DOMC data, we consider this most restrictive model as Model 1, the most general model in (3) as Model 2, and the model with *a*_*i*(*k*)_ = *a*_*i*_ as Model 3.

Our expectation is that the LPE will emerge as statistically superior to each of Models 1–3. Specifically, the repeated administration of response options seemingly maps closely to the notion of distinct item steps that motivate the model. Moreover, due to the random administration of response options, we can attach a psychometric equivalence to each of the steps, as each option as has equal likelihood of emerging at each step. We consider two versions of the LPE. The first, based on Equations (1) and (2), assumes for each item a unique *a*_*i*_ and *b*_*i*_ that remain constant across the scheduled number of response options *k*, but an exponent parameter ξ_*ik*_ that varies as a function of item *i* and *k*. We denote this model as Model 4. However, it might be anticipated that to the extent that each added response option may have a consistent effect on the change in the ξ parameter within an item, we could also consider a constrained model in which the ξ parameter changes as a linear function of the number of scheduled response options, i.e., ξ_*ik*_ = *c*_i_^*^ *k*, and thus only a single *c*_*i*_ parameter is estimated in relation to the exponent of each item. We consider this constrained version of Model 4 as Model 5.

## Model estimation and comparison

Various approaches to the estimation of the LPE have been presented, although the most promising appear to be those based on Markov chain Monte Carlo (MCMC; see Bolfarine and Bazán, 2010). As such an approach can be easily adapted in estimating the comparison 2PL models, we also apply it in this paper using WINBUGS 1.4 (Spiegelhalter et al., 2003). Adopting the same priors as in Bolfarine and Bazán (2010), for Model 4 we specify item parameters for each item as

$$\begin{array}{l} \xi_{ik}\sim Gamma\;(0.25,\;0.25),\\ \;b_{i}\sim Normal\;(0,\;1.0),\\ \;a_{i}\sim Lognormal\;(0,\;0.5) \end{array}$$

while for person parameters,

$$\begin{array}{l} \theta_{j}\sim Normal\;\left(\text{0, 1.0}\right). \end{array}$$

For the special case of Model 5, we specified

$$\begin{array}{l} \xi_{i}\sim Gamma\;\left(\text{0.25, 0.25}\right) \end{array}$$

while the constant within-item slope that reflects the parameter change in relation to scheduled number of response options (i.e., ξ_*ik*_ = *c*_i_^*^ *k*) is given by

$$\begin{array}{l} c_{i}\sim Gamma\;\left(\text{1, 2}\right). \end{array}$$

Each of the 2PL models considered (Models 1, 2, and 3 above) were specified using the same corresponding prior distributions so as to facilitate comparison of the models.

Markov chains were simulated up to 10,000 iterations, and parameter estimates were based on the posterior means. Convergence of the chains was evaluated using the Gelman-Rubin criterion following a simultaneous simulation of four additional chains for each model.

Table 2 displays the results in terms of model comparison with respect to the Deviance Information Criterion (DIC; Spiegelhalter et al., 2002). Following the DIC criterion, the preferred model is that with lowest DIC. In this regard, we find Model 4, which hold *a* and *b* constant within an item but allows the ξ parameter to vary with respect to *k*, to be the preferred model. In particular, Model 4 provides a superior fit to Model 2, which varies the *a* and *b* parameters as a function of *k*. This finding suggests that manipulation of the ξ parameter provides (as anticipated) a useful way of accommodating effects of key location, and is more economical than allowing both the *a* and *b* parameters to vary. However, the assumption of a linear change in the exponent in relation to *k* is not supported, as Model 4 appears superior to Model 5.

**Table 2:** Empirical comparison of IRT models applied to IT certification data (*N* = 648).

	**Model**	**D-Bar**	**D-Hat**	**pD**	**DIC**
1	2PL × Item	43874.8	43142.7	732.2	44,607
2	2PL × Item × #RespOpt (a and b)	40681.8	39573.7	1108.1	41,790
3	2PL × Item × #RespOpt (b only)	40938.9	39985.2	953.6	41,893
4	LPE × Item × #RespOpt (ξ_*i*_ only)	40798.8	40119.8	678.9	41,478
5	LPE × Item (ξ as a linear function of #RespOpt)	40868.0	40185.9	682.1	41,550

Table 3 displays the distribution of ξ estimates in relation to the scheduled number of response options for single-keyed, double-keyed, and triple-keyed items. Despite the inferior fit of Model 5 above, the change in ξ across the number of scheduled response options appears to occur on average in approximately equal intervals.

**Table 3:** Mean (Standard Deviation) of ξ estimates in relation to the number of scheduled response options.

**# RespOpt**	**Single keyed items**	**Double keyed items**	**Triple keyed items**
1	0.619 (0.417)		
2	1.000 (0.485)	1.013 (0.460)	
3	1.487 (0.603)	1.629 (0.610)	1.468 (0.379)
4	1.819 (0.703)	2.246 (0.717)	1.832 (0.285)
5			2.534 (0.801)

It is conceivable that despite these nearly equal intervals, the effect of the number of scheduled response options could have a non-linear effect within individual items. Factors that could explain such results could be effects related to the differential attractiveness of keyed vs. distractor options, or alternatively, examinee expectations in terms of the number of keyed responses per item.

## Illustration of example items

**Figure 2:**
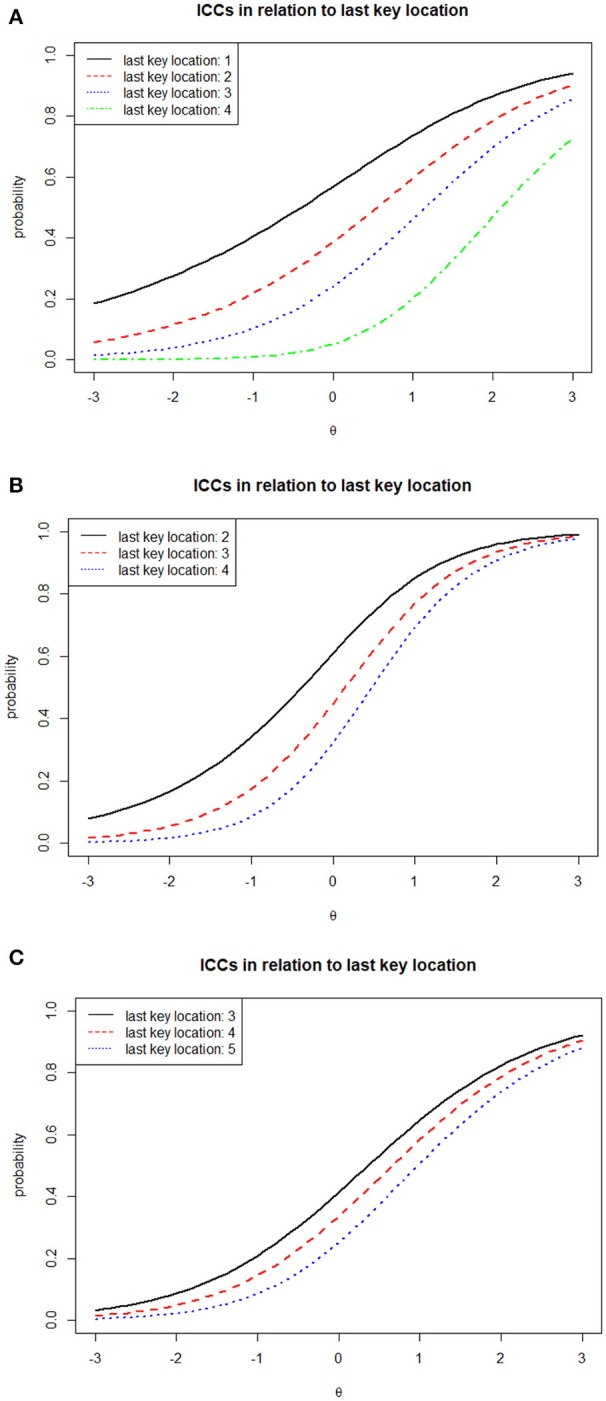
Estimated item characteristic curves (ICCs) for three DOMC items, IT Certification Test (*N* = 648). **(A)** Single-Keyed Item (1–4 Possible Response Options); Item 18 (a = 0.953, b = 1.007, ξ_1_ = 0.441, ξ_2_ = 0.742, ξ_3_ = 1.107, ξ_4_ = 2.303). **(B)** Double-Keyed Item (2–4 Possible Response Options); Item 30 (a = 1.461, b = 0.307, ξ_1_ = 0.526, ξ_2_ = 0.850, ξ_3_ = 1.198). **(C)** Triple-Keyed Item (3–5 Possible Response Options); Item 27 (a = 0.913, b = 0.123, ξ_1_ = 1.176, ξ_2_ = 1.450; ξ_3_ = 1.833).

Figure 2 illustrates ICCs of several of the DOMC items and their variability as a function of the scheduled number of response options. Items 18, 30, and 27 represent examples of single-keyed, double-keyed, and triple-keyed items. From the earlier description, for such item types there are 1–4, 2–4, and 3–5 possible response options, respectively, implying 4, 3, and 3 ξ parameters for each item for each respective type. Although independently estimated, we naturally expect the ξ estimates to increase within each item as the number of scheduled response options increase. This is true for the three items shown here, and in fact was true for all 84 items included in the analysis. The corresponding curves illustrate the model-based functioning of the items across the different numbers of scheduled options. As implied earlier, we consistently see the curves move to the right (implying increased difficulty) and become steeper at the inflection point (implying increased discrimination) as the last key location (or equivalently, the scheduled number of response options) increases. At the same time, the effect of the scheduled number of response options on item functioning also varies quite considerably. For example, in item 18 the curves are quite far apart, while for Item 27 they are much closer together. Such effects may well reflect variability in the overall attractiveness of distractor options. Effective distractors will naturally tend to make the curves spread out more, while ineffective distractors will keep them close together.

## Implications for IRT metric

As noted earlier, one practical consequence of the use of asymmetric IRT models concerns its effect on the latent IRT metric. Bolt et al. (2014) illustrated by simulation how the presence of asymmetry (as caused by variability in item complexity) may induce a shrinkage in the latent metric especially at the higher end of the proficiency scale when traditional symmetric IRT models are fit instead. Specifically, in the presence of positive asymmetry (as would caused by items that have ξ > 1), it is by pulling the units of the latent metric at the upper end of the scale closer to the mean that a symmetric model such as the 2PL can be made to fit. To the extent that the latent metrics in IRT are often viewed (and treated) as having interval level properties, the shrinkage is consequential in practice. Failure to appropriately attend to the asymmetry of the curves (as supported by the model comparison criteria) can among other things make it more difficult for high ability examinees to demonstrate gains from one administration of a test to the next.

**Figure 3:**
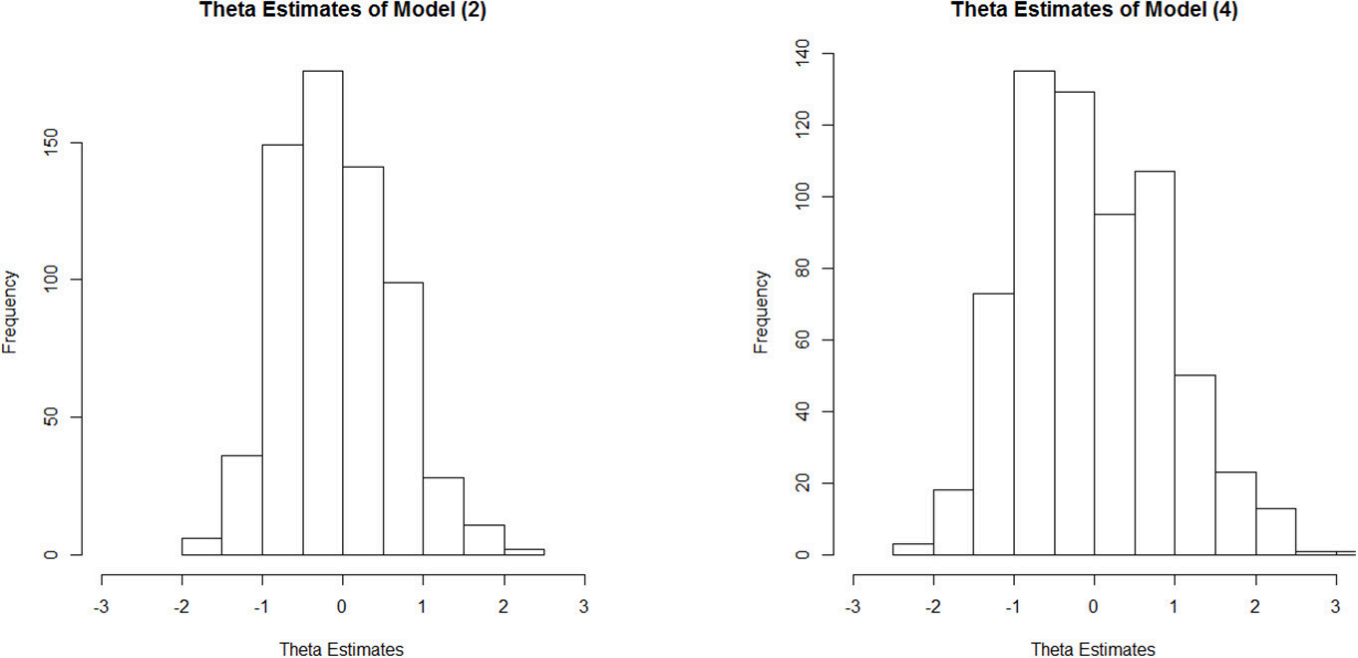
Histograms of proficiency estimates under Model 2 (symmetric 2PL model) and Model 4 (asymmetric LPE model).

We can illustrate this occurrence in relation to the DOMC data by contrasting the latent metric that emerges under Model 4 vs. that seen under Model 2. Figure 3 provides a histogram of the proficiency (θ) estimates for the 648 examinees as estimated under each model. Despite both calibrations scaling the θ metric to have a mean of 0 and standard deviation of 1, under Model 4 we see greater spread in the actual estimates, especially at higher proficiency levels.

The cause of the shrinkage in this context can be recognized by appealing to Figure 1. When multiple items of the type with ξ > 1 are fit using symmetric IRT models, the tendency for more difficult items to be positively asymmetric results in the latent proficiency units being pulled closer to 0 so as to render a symmetric curve. Further details are provided by Bolt et al. (2014) and illustrated using simulation. The results here further confirm the effect now using real test data.

## Conclusion and discussion

Our results support the potential benefit of an LPE model in the scoring of test performances using DOMC. For DOMC applications, the LPE has appeal for both psychological and statistical reasons. As a psychological model, the LPE provides a natural mechanism by which multiple conjunctively interacting subprocesses (in this case, the independent responses to different response options) are captured through an exponent parameter. Our findings aligned with expectations in that the increased difficulty and discrimination seen as the last key is located later also corresponded to systematic increases in the ξ parameter estimates. This approach also provides statistical advantages in the sense that we are able to simultaneously account for the difficulty and discrimination effects of key location through one (as opposed to two) added parameter for each potential key location. Further, by accounting for the asymmetry of ICCs, we are able to remove shrinkage in the higher end of the latent proficiency scale, yielding a metric that will be more sensitive to student differences (and thus more suitable for demonstrating growth) at higher proficiency levels.

In a broader sense, our results also extend Lee and Bolt (2018a) in showing that asymmetric IRT models have the potential to function as useful validation tools in educational measurement. The DOMC items provided a useful context in which to explore this theory due to the administration of DOMC items in a random fashion that varies the number of response options examinees must respond to in answering the item correctly. Under the DOMC format, each additionally presented response option naturally implies an additional “step” in solving the item. As noted, we view the DOMC format as a natural experiment for demonstrating how aspects of response process can emerge through LPE parameter estimates, in this case the exponent parameter ξ.

We believe that additional psychometric study of asymmetry in IRT models is warranted. Such research can focus not only on the models themselves, but also the consequences of ignoring asymmetry when present. As assessments seek deliberately to incorporate items of greater complexity, and as computer-based assessments open the door to unique item types such as DOMC that have the potential for greater complexity, models that attend more closely to response process likely will become increasingly important. There of course also remain many potential additional directions of research with DOMC items, including the potential for closer comparisons against traditional multiple-choice.

## Author contributions

DB, SL, JW, and CE contributed to conception and design of the study. JS organized the database. DB and SL performed the statistical analysis. DB wrote the first draft of the manuscript. SL, JW, and CE wrote sections of the manuscript. All authors contributed to manuscript revision, read and approved the submitted version.

### Conflict of interest statement

The authors declare that the research was conducted in the absence of any commercial or financial relationships that could be construed as a potential conflict of interest.
